# Impact of syringaldehyde on the growth of *Clostridium beijerinckii* NCIMB 8052 and butanol production

**DOI:** 10.1007/s13205-011-0042-4

**Published:** 2012-01-03

**Authors:** Catherine Richmond, Victor Ujor, Thaddeus Chukwuemeka Ezeji

**Affiliations:** Department of Animal Sciences, Ohio State Agricultural Research and Development Center (OARDC), The Ohio State University, 305 Gerlaugh Hall, 1680 Madison Avenue, Wooster, OH 44691 USA

**Keywords:** *Clostridium beijerinckii*, Butanol, Acetone, Syringaldehyde, Coenzyme A transferase, CoAT

## Abstract

While lignocellulosic biomass excels as a cheap, renewable resource for biofuel production, it does present some challenges such as generation of microbial inhibitory compounds. The mode of selective inhibition of acetone–butanol–ethanol (ABE) production (as opposed to cell growth) by syringaldehyde on *Clostridium beijerinckii* NCIMB 8052 was examined. *C. beijerinckii* 8052 grown in syringaldehyde-supplemented P2 medium had a comparable growth rate (μ = 0.34) at acidogenic growth phase to that of *C. beijerinckii* 8052 grown in control P2 medium (μ = 0.30). The addition of syringaldehyde into P2 medium inhibited solvent production by *C. beijerinckii* 8052 and increased butyric and acetic acid accumulation in the fermentation broth. Analysis of coenzyme A transferase (CoAT) using cell-free extracts of *C. beijerinckii* 8052 showed decreased expression and activity in the presence of syringaldehyde. These results indicate that *C. beijerinckii* 8052 CoAT is negatively affected by syringaldehyde and thus, hampers the ability of the microorganism to metabolize butyric and acetic acid for ABE production as evidenced by the accumulation of butyric and acetic acid in the fermentation broth.

## Introduction

The production of acetone, butanol and ethanol (typically referred to ABE) by means of solventogenic *Clostridium* species used to be one of the largest industrial fermentation processes; second to only ethanol fermentation in the early 1900 s (Jones and Woods 1986). Solventogenic *Clostridium* species undergoes a biphasic fermentation in which acetic and butyric acid are predominantly produced during the exponential growth phase (acidogenic stage, 3–18 h) followed by re-assimilation of acid for the production of ABE during late exponential and stationary growth phases (solventogenic stage, >15 h) (Andersch et al. 1983; Dürre 2008; Ezeji et al. 2010). Butanol, for instance, can be used as fuel in some vehicles without the need to modify internal combustion engines, which is necessary in those cases where ethanol is used as fuel (Antoni et al. 2007).

The commonest substrate used today for biofuel (ethanol and butanol) production is corn. Solventogenic *Clostridium* species secrete numerous enzymes such as α-amylase, α-glucosidase, glucoamylase, pullulanase, and amylopullulanase that facilitate the breakdown of corn (starch) into glucose prior to fermentation of glucose to ABE (Ezeji et al. 2007a). Although corn is renewable, its use as a primary feed for livestock and other consumer products creates competition in the market and drives up the cost of corn (Yacobucci and Schnepf 2007). More economical choices are non-food crop renewable lignocellulosic substrates that are generally considered as waste, such as wood shavings, sawdust, corn stover, wheat straw, sugarcane bagasse, and other lignocellulosic biomass from agricultural residues.

Solventogenic *Clostridium* species are unable to hydrolyze lignocellulosic biomass into monomeric sugars prior to ABE fermentation. Pretreatment and hydrolysis of lignocellulosic biomass is required to allow the bacteria access to the sugars inside the lignin and hemicellulose matrix of the biomass. Unfortunately, the pretreatment process also releases inhibitory compounds, such as syringaldehyde, ρ-coumaric acid, furfural, 5-hydroxymethyl furfural (HMF), ferulic acid, vanillin, and phenols, which inhibit the growth and solvent production by solventogenic *Clostridium* species (Ezeji et al. 2007b). It is important to note that the hemicellulose component of lignocellulosic biomass contains acetate and glucuronate, which upon hydrolysis are released into solution as acetic and glucuronic acid resulting in the acidification of lignocellulosic hydrolysates (pH < 4.0). The low pH of the hydrolysates in conjunction with high temperatures triggers dehydration of sugars and depolymerization of lignin to microbial inhibitory compounds (Ezeji et al. 2007a, b). The process to remove these inhibitors from hydrolysates is cost-prohibitive (Almeida et al. 2009). To use solventogenic *Clostridium* species to convert lignocellulosic biomass hydrolysates into butanol, they must be able to grow and produce butanol in the presence of the released microbial inhibitors. Understanding how these inhibitors affect solventogenic *Clostridium* species will allow further work to be done in developing inhibitor-tolerant strains.

One inhibitor of particular interest is syringaldehyde. Syringaldehyde is an aromatic aldehyde released during the chemical hydrolysis of lignocellulosic biomass, specifically from partial breakdown of lignin through syringyl monomer units (Olsson and Hahn-Hägerdal 1996) and exerts inhibitory effects on microbial growth. Various concentrations of syringaldehyde comprising 0.24–1.82 mg/L hydrolysates (Cantarella et al. 2006), 6–14 mg/g biomass (Chen et al. 2006), and 0.95 mg/g ammonium fiber expansion (AFEX) pretreated biomass (Balan et al. 2009) have been reported. Depending on the lignocellulosic biomass (plant) species, age of maturity and the type of pretreatment employed, the concentration of syringaldehyde in lignocellulosic hydrolysates can range from 0.3 to 2 g/L (Fonseca et al. 2011). While most inhibitory hydrolysates such as vanillin, furfural, HMF, acetic acid, ferulic acid, glucuronic acid, and ρ-coumaric acid (Olsson and Hahn-Hägerdal 1996; Ezeji et al. 2007b) cause a decrease in bacterial growth ensuing in decreased solvent production, syringaldehyde specifically inhibits ABE formation in solventogenic *Clostridium* species. The mode of selective inhibition by syringaldehyde on ABE production (as opposed to cell growth) by *Clostridium beijerinckii* NCIMB 8052 was examined. This study describes the cultivation of *C. beijerinckii* NCIMB 8052 in syringaldehyde-supplemented P2 medium (treatments) and examines the effect of syringaldehyde on *C. beijerinckii* NCIMB 8052 growth rate, generation time, and coenzyme A transferase (CoAT). *C. beijerinckii* NCIMB 8052 is capable of converting fermentation intermediates (acetic and butyric acid) to butanol and acetone because it possesses CoAT (Wiesenborn et al. 1989; Tummala et al. 2003; Ezeji et al. 2010; Han et al. 2011).

## Materials and methods

### Microorganism, batch fermentation and inhibition studies

*C. beijerinckii* NCIMB 8052 (ATCC 51743) was obtained from American Type Culture Collection, Manassas, VA, USA, and used for this study. Culturing methods and use of syringaldehyde for inhibition studies were performed as follows: 300 μL of *C. beijerinckii* NCIMB 8052 spores previously stored in sterile double-distilled water at 4 °C was heat-shocked at 75 °C for 10 min, cooled on ice for 5 min, and then transferred into 10 mL tryptone glucose yeast extract (TGY) medium to incubate at approximately 35 °C overnight in an anaerobic chamber (Coy, Ann Arbor, MI). The anaerobic chamber had a modified atmosphere of 82% N_2_, 15% CO_2_, and 3% H_2_. Cultures were checked between 12 and 13 h to determine the growth based on optical density (OD_540_) measurements with a DU 800 spectrophotometer (Beckman Coulter Inc, Brea, CA). This was followed by transferring 10 mL of actively (OD 0.8–1.0 at 540 nm) growing *C. beijerinckii* NCIMB 8052 into 90 mL TGY medium. *C. beijerinckii* NCIMB 8052 cells were incubated anaerobically at 35 ± 1 °C for 3–5 h until the OD_540_ was between 0.8 and 1.0 before 6 mL of the culture is transferred into 91 mL sterile butanol production P2 medium (60 g/L glucose and 1 g/L yeast extract). To evaluate the effect of syringaldehyde on *C. beijerinckii* NCIMB 8052 cells, 0.2–1.0 g/L syringaldehyde was added into P2 medium prior to sterilization by autoclaving (15 min at 121 °C). Prior to the inoculation of P2 medium or syringaldehyde-supplemented P2 medium with *C. beijerinckii* NCIMB 8052 cells, the medium was supplemented with 1 mL each of filter-sterilized P2 stock vitamin (*p*-aminobenzoic acid 0.1 g/L, thiamine 0.1 g/L, biotin 0.001 g/L), mineral (MgSO_4_7H_2_O 20 g/L, MnSO_4_H_2_O 1.0 g/L, FeSO_47_H_2_O 1.0 g/L, NaCl, 1.0 g/L), and buffer (KH_2_PO_4_ 50 g/L, K_2_HPO_4_ 50 g/L, CH_3_COONH_4_ 220 g/L) solutions, each equaling 1% of the total fermentation volume (Ezeji et al. 2003, 2004). The fermentation was allowed to proceed for 72–84 h and samples were collected at 3 or 12 h intervals for analysis.

### Determination of growth rate and generation time of *C. beijerinckii*

*C. beijerinckii* 8052 was grown in the anaerobic chamber for 72 h following the procedure described in the previous section on “Microorganism, batch fermentation and inhibition studies”. Samples were aseptically collected at 3 h interval for 36 h for growth analysis. Growth of *C. beijerinckii* 8052 was estimated by measuring optical density (OD_540_ nm λ) using a DU800 spectrophotometer (Beckman Coulter Inc., Brea, CA, USA). Cell dry weight was determined by employing a predetermined correlation between optical density and cell dry weight as described previously (Ezeji et al. 2003). Cell dry weight (g/L) of *C. beijerinckii* 8052 was used to calculate growth rates and generation times. The generation time (*g*) was calculated as follows: *g* = [(log *W*_t_)−(log *W*_0_)]/log2; where *W*_0_ is weight of cells at initial/zero time and *W*_t_ is weight of cells at final time. The growth rate (μ) was determined using the equation μ = [(ln *W*_t_)−(ln *W*_0_)]/(*t*_t_−*t*_0_); where *t*_0_ is initial/zero time and *t*_1_ is final time.

Growth of *C. beijerinckii* 8052 and ABE fermentation was allowed to proceed for 84 h after which the fermentation self terminated.

### Effect of syringaldehyde on Coenzyme A transferase (CoAT) activity by *C. beijerinckii* 8052

Equal amounts (2 mg dry weight) of *C. beijerinckii* 8052 cells grown in P2 medium (control) or *C. beijerinckii* 8052 grown in syringaldehyde-supplemented P2 medium (1.0 g/L syringaldehyde) (treatment) following 24 and 36 h of fermentation which represent the early and late solventogenic growth phase, respectively, were collected. Cell pellets were stored at −70 °C until subsequent use. Frozen cells were thawed on ice, followed by suspension of the cell pellet in 500 μL of 50 mM MOPS [3-(*N*-morpholino) propanesulfonate], 500 mM ammonium sulfate, 20% glycerol and 2 μg lysozyme. The mixture was incubated at 35 °C for 2 h. *C. beijerinckii* cells were lysed with TissueLyser LT (Qiagen Inc., Valencia, CA, USA) to obtain cell-free extract as previously described (Han et al. 2011). These extracts were transferred into new sterile tubes and were used either immediately or stored at −70 °C prior to enzyme activity assays. Two hundred microliters of the cell-free extract was used for CoAT assay as described previously (Clark et al. 1989; Chen and Blaschek 1999). We used an acetoacetyl-CoA extinction coefficient of 8 mM^−1^cm^−1^ at Abs_310._ Two hundred microliters of cell-free extract of *C. beijerinckii* 8052 was used per 1 mL assay volume. One unit of CoAT activity is defined as the conversion of 1 μmol of acetoacetyl-CoA to acetoacetate per min per mg protein. The equation to determine the enzyme activity level in relation to *C. beijerinckii* 8052 cell amount (a) was: *a* = (μmol/min × dilution factor)/*g* of cells in crude cell extract sample.

### Effect of syringaldehyde on Coenzyme A transferase (CoAT) activity of *C. beijerinckii* 8052

Two hundred microliters of cell-free extracts of *C. beijerinckii 8052* grown in P2 medium or in syringaldehyde-supplemented P2 medium was used to determine CoAT activity (Clark et al. 1989; Chen and Blaschek, 1999) in the presence of 1.0 g/L syringaldehyde (treatment) and in the absence of syringaldehyde (Control).

### Fermentation parameters and analysis

Residual concentrations of syringaldehyde in the sample was measured by HPLC equipped with a photodiode array (PDA) detector (Waters, Milford, MA) and a 3.5 μm Xbridge C18, 150 × 4.6-mm column (Waters, Milford, MA) as described previously (Matějíček et al. 2003). Samples were filtered through 0.45-μm syringe filters prior to injections. Acetone, butanol, ethanol, acetic and butyric acid concentrations were measured using a 7890A Agilent Technologies gas chromatograph (Agilent Technologies, Santa Clara, CA, USA) equipped with a flame ionization detector (FID) and a 30 m (length) × 320 μm (internal diameter) × 0.50 μm (HP-Innowax film) J × W 19091 N-213 capillary column (Han et al. 2011). Productivity of ABE was calculated as total concentration (g/L) divided by fermentation time (h). Yield was defined as total grams of ABE produced per total grams of glucose utilized.

Glucose concentrations were determined using a hexokinase and glucose-6-phosphate dehydrogenase coupled enzymatic assay (Ezeji et al. 2003). Crude protein concentrations of the cell-free extracts of *C. beijerinckii* were determined using the Bradford protein assay method as previously described (Ezeji and Bahl 2006).

### Statistical analysis

Multiple one-way analyses of variance (ANOVA) were conducted to investigate the effect of syringaldehyde on growth, acetic and butyric acid production, crude proteins expressions, CoAT activities, butanol and ABE production. Syringaldehyde was used as independent variable while butanol and ABE concentration, cell growth, acetic and butyric acid production, CoAT expression and activities were considered dependent variables. Unless otherwise stated, all results were expressed as mean ± SD (*n* ≥ 3). Tukey’s adjustment was applied to the pair-wise comparisons to determine significant difference (*P* < 0.05) among treatments. All analyses were conducted using the General Linear Model (GLM) procedure of SAS Version 9.1.3 (SAS Institute Inc., Cary, NC, USA).

## Results and discussion

### Effect of different concentrations of syringaldehyde on the growth of and glucose utilization by *C. beijerinckii*

**Fig. 1:**
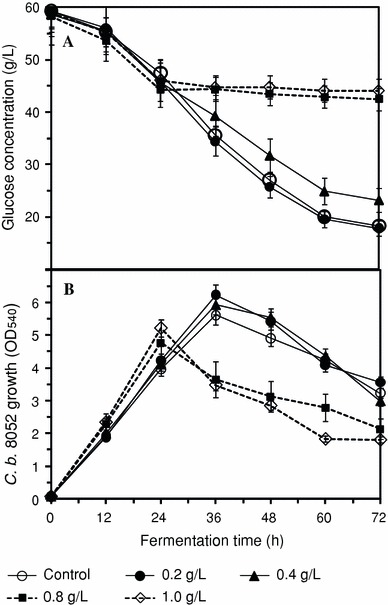
Kinetic behavior of glucose utilization (**a**) and cell growth (**b**) by *C. beijerinckii* NCIMB 8052 in P2 medium supplemented with different (0.2–1.0 g/L) concentrations of syringaldehyde. Data represent averages of results from at least three fermentations

The initial sugar level in the growth medium was 59 g/L in all treatments. Figure 1a presents the time course of glucose utilization during the growth of *C. beijerinckii* in P2 medium containing different concentrations of syringaldehyde. Interestingly, syringaldehyde (0.2–1.0 g/L) enhanced the growth of *C. beijerinckii* by 4–31% during initial 24 h of fermentation when compared to the control experiment (Fig. 1b). In contrast, syringaldehyde has been shown to reduce the growth of *E. coli* KO11 and *E. coli* LY01 by more than 75% during the first 24-h postinoculation and by 40% after 48 h of growth (Zaldivar et al. 1999). Prior reports have shown that syringaldehyde (≤2.0 g/L) had no significant effect on the overall growth of *C. beijerinckii* BA101 (Ezeji et al. 2007b). In view of the biphasic (acidogenic and solventogenic) life cycle nature of solventogenic *Clostridium* species, however, it is not clear whether syringaldehyde will have profound impact at a particular growth phase. Analysis of the impact of syringaldehyde on *C. beijerinckii* 8052 growth and generation time revealed that *C. beijerinckii* 8052 had a growth rate, μ, of 0.34 and a generation time of 2.21 h in the presence of 1.0 g/L syringaldehyde at the exponential growth phase during which acetic and butyric acid are produced predominantly (Table 1). Similarly, average generation time of 2.46 h and growth rate of 0.30 were obtained with control fermentations (Table 1). Consequently, the presence of syringaldehyde in the fermentation medium did not have significant effect on the generation time and growth rate of *C. beijerinckii* 8052 during exponential growth phase. As expected, the growth rate of *C. beijerinckii* 8052 grown in both P2 control and syringaldehyde-supplemented P2 media decreased drastically (Table 1) at the solventogenic growth phase during which previously produced acetic and butyric acid were expected to be re-assimilated for ABE production. Although the presence of syringaldehyde marginally increased the growth rate of *C. beijerinckii* 8052 with concomitant decrease in generation time during exponential phase, the growth rate of *C. beijerinckii* 8052 during the solventogenic growth phase in P2 control medium was 117% more than that of the syringaldehyde-supplemented P2 medium (Table 1). At the end of fermentation residual glucose concentration was 18.3 g/L for the control fermentation. It can be noted that the utilization of glucose by *C. beijerinckii*, as estimated by measurement of residual glucose concentration after fermentation, varied (17.8–44.1 g/L) during growth in syringaldehyde-supplemented P2 medium. The reduction (13–64%) in glucose utilization by *C. beijerinckii* during growth in syringaldehyde-supplemented P2 medium (>0.4 g/L syringaldehyde) is associated with abrupt decrease in growth after 24 h of the fermentation (Fig. 1). In contrast, the growth of *C. beijerinckii* in P2 medium (control) or syringaldehyde-supplemented P2 medium (≤0.4 g/L syringaldehyde) extended to 36 h after which gradual decrease ensued (Fig. 1b).

**Table 1:** Mean generation times and growth rates (μ) of *C. beijerinckii* NCIMB 8052 in the presence and the absence of syringaldehyde

Fermentation treatments	*Generation time (h)	^§^Growth rate (μ)	
		Acidogenic phase	Solventogenic phase
Control (P2 medium)	2.46^a^ ± 0.67	0.30^b^ ± 0.08	0.013^c^ ± 0.005
Syringaldehyde-supplemented P2 medium	2.21^a^ ± 0.53	0.34^b^ ± 0.08	0.006^d^ ± 0.001

*^,§^ Mean values with the same superscript letter are not significantly different

### Acetic and butyric acid production and re-assimilation by *C. beijerinckii* 8052

**Fig. 2:**
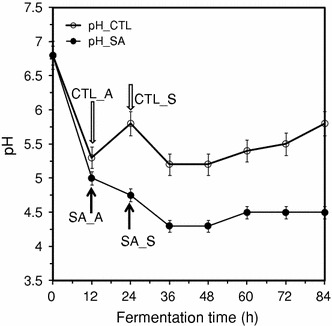
pH variations during growth of *C. beijerinckii* NCIMB 8052 in syringaldehyde-supplemented P2 medium (1.0 g/L syringaldehyde). CTL and SA represent the control and syringaldehyde-supplemented P2 medium. CTL_A and CTL_S represent pH of the control fermentation at acidogenic and solventogenic phases, respectively. *First arrow* shows lowest pH reached during acidogenic phase. *Second arrow* shows increase in pH due to switch to solventogenic phase (control) and further decrease in pH due to inability to switch to solventogenic phase (treatment). Data represent averages of results from at least three fermentations

**Fig. 3:**
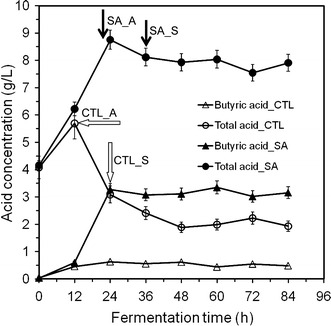
Butyric acid and total acid production by *C. beijerinckii* NCIMB 8052 in syringaldehyde-supplemented P2 medium (1.0 g/L syringaldehyde). CTL and SA represent the control and syringaldehyde-supplemented P2 medium. CTL_A (*first arrow*) and CTL_S (*second arrow*) represent acid production by *C. beijerinckii* grown in P2 medium at the peak of acidogenic and early solventogenic phase, respectively. SA_A (*first arrow*) and SA_S (*second arrow*) represent acid production by *C. beijerinckii* grown in syringaldehyde-supplemented P2 medium at the peak of acidogenic and early solventogenic phase, respectively. *Second arrow* shows marked decrease in acid concentration due to switch to solventogenic phase (control) and negligible decrease in acid concentration due to inability to switch to solventogenic phase (treatment). Note that gas chromatography recognizes and measures both acetic acid and acetate (component of P2 medium) as same compound. This fact explains why up to 4 g/L acid was measured at 0 h with an initial pH of 6.8

Acetic and butyric acid production by *C. beijerinckii* during the acidogenic growth phase and their re-assimilation (solventogenic growth phase) are essential for butanol production and longevity of solventogenic *Clostridium* species during ABE fermentation (Ezeji et al. 2010). An initial drop in pH following an increase of acetic and butyric acid production during the acidogenic growth phase is evident at 12 h for both control and treatment fermentations (Fig. 2). While the pH profile of *C. beijerinckii* 8052 grown in P2 medium shows the typical biphasic fermentation (Ezeji et al. 2010), the pH profile of *C. beijerinckii* grown in syringaldehyde-supplemented P2 medium (1.0 g/L syringaldehyde) revealed a continuous decrease in pH falling to below pH 4.5 (Fig. 2). Acetic and butyric acid analysis showed that acid concentration in the fermentation medium reached 5.6 g/L (CTL_A) in 12 h prior to solventogenesis (CTL_S) during which a sharp decrease in acetic and butyric acid concentration occurred (Fig. 3; CTL_S) due to acid uptake and conversion to ABE. In contrast, *C. beijerinckii* grown in syringaldehyde-supplemented P2 medium accumulated up to 8.7 g/L acid (SA_A) in 24 h without marked decrease in acid concentration after 24 h (Fig. 3) due to an apparent failure of a “switch” from acidogenic to solventogenic stage culture, a phenomenon known as “acid crash”, which occasionally occurs in ABE fermentations. “Acid crash” occurs when the acid concentration in the fermentation broth exceeds the maximum tolerable limit or formic acid is produced, causing cessation of glucose uptake and rapid termination of solventogenesis (Maddox et al. 2000; Wang et al. 2011). It is worth mentioning that more than 3 g/L butyric acid, about seven times greater than that obtained from the control medium, accumulated in the fermentation medium during growth of *C. beijerinckii* in syringaldehyde-supplemented P2 medium (Fig. 3). Butyric acid inhibits microbial cells by disrupting cell membrane functions (Martin et al. 1983; Alsaker et al. 2009). Toxicity of butyric acid to solventogenic *Clostridium* species, in addition, increases as the pH decreases from 6.0 to 4.0 (Monot et al. 1984). Growth of *C. beijerinckii* in the syringaldehyde-supplemented P2 medium resulted in a pH below 4.5 (Fig. 2) and accumulation of toxic levels of butyric acid in the fermentation medium (Fig. 3). These data suggest that acid crash may be the real cause for the premature cessation of *C. beijerinckii* growth following 24-h fermentation and termination of the fermentation process.

**Fig. 4:**
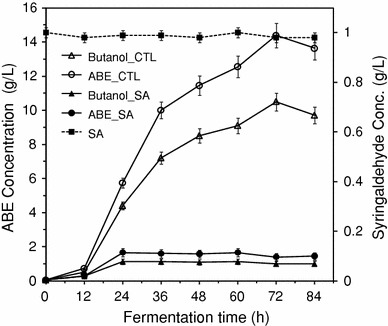
Butanol and total ABE production by *C. beijerinckii* grown in P2 medium and syringaldehyde-supplemented P2 medium. SA with *broken lines* represents concentration of syringaldehyde during the course of fermentation. Butanol_CTL and ABE_CTL represent butanol and total ABE production by *C. beijerinckii* grown in P2 medium. Butanol_SA and ABE_SA represent butanol and total ABE production by *C. beijerinckii* grown in syringaldehyde-supplemented P2 medium. Data represent averages of results from at least three fermentations

Cofactors (NADH and NADPH) are involved in the detoxification of aldehydes by microorganisms (Miller et al. 2009). These cofactors are also involved in butanol synthesis and play vital role during transition from acidogenesis to solventogenesis (Grupe and Gottschalk 1992). It is conceivable that NADH and NADPH pools did not reach levels required for sustenance of glycolysis due to diversion to syringaldehyde detoxification, resulting in inability of *C. beijerinckii* to transition from acidogenesis to solventogenesis (Zhang et al. 2011). To test this hypothesis, syringaldehyde concentration in the syringaldehyde-supplemented P2 fermentation medium was analyzed. Figure 4 indicates that *C. beijerinckii* cells grown in syringaldehyde-supplemented P2 medium did not metabolize syringaldehyde but butanol production was negatively affected. Therefore, the failure of *C. beijerinckii* to transition from acidogenic to solventogenic phase was not as a result of depletion of cofactors pool due to syringaldehyde detoxification.

### Effect of syringaldehyde on ABE production by *C. beijerinckii* NCIMB 8052

To understand the effect of syringaldehyde on ABE production, samples were taken every 12 h and tested for acetone, butanol, and ethanol. The ABE production profiles by *C. beijerinckii* using P2 medium over the course of 84 h showed that the culture produced 10.5 g/L butanol, 3.4 g/L acetone, and 0.5 g/L ethanol resulting in a total ABE concentration of 14.4 g/L (Fig. 4). In contrast, when *C. beijerinckii* was grown in syringaldehyde-supplemented P2 medium, only 1.1 g/L butanol, 0.3 g/L acetone, and 0.3 g/L ethanol resulting in a total ABE concentration of 1.7 g/L was produced (Fig. 4). Considering the fact that a maximum OD_540_ of 5.22 was obtained in 24 h for *C. beijerinckii* grown in syringaldehyde-supplemented P2 medium compared to OD_540_ of 5.93 obtained in 36 h for control fermentation (Fig. 1b), the 1.1 g/L butanol produced in the presence of syringaldehyde (Fig. 4) suggests that processes involved in the synthesis of ABE in *C. beijerinckii* was negatively affected by syringaldehyde. To determine why the growth of *C. beijerinckii* in syringaldehyde-supplemented P2 medium resulted in pH below 4.5 (Fig. 2) and accumulation of toxic levels of butyric acid in the fermentation medium (Fig. 3), we investigated the effect of syringaldehyde on protein and CoAT expressions in *C. beijerinckii*.

**Fig. 5:**
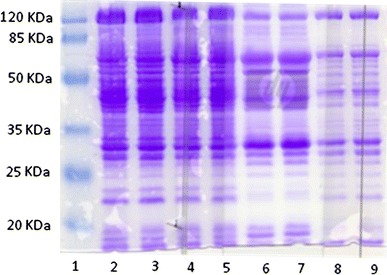
Representative 12% SDS-PAGE of *C. beijerinckii* 8052 protein extracts stained with Coomassie blue R-250. *Lane 1*: protein ladder; *lanes 2* and *4*: crude protein profile of *C. beijerinckii* during exponential growth phase (fermentation time, 12 h) in P2 medium (control); *lanes 3* and *5*: crude protein profile of *C. beijerinckii* during acidogenic growth phase (fermentation time, 12 h) in P2 medium containing syringaldehyde (1 g/L); *lanes 6* and *7*: crude protein profile during solventogenic growth phase (fermentation time, 24 h) in P2 medium; *lanes 8* and *9*: crude protein profile during solventogenic growth phase (fermentation time, 24 h) in P2 medium containing syringaldehyde (1 g/L). Test and control cultures were adjusted to an OD600 ≈1.0 at 12 and 24 h and cell pellets from 2 mL samples were lysed with tissue lyser (Qiagen, Hilden Germany) for protein extraction. Fewer proteins from solventogenic growing culture of *C. beijerinckii* were used for the SDS PAGE

Total protein from equal amounts (5.8 mg dry weight) of *C. beijerinckii* cells grown in P2 and syringaldehyde-supplemented P2 medium media were extracted during acidogenic and solventogenic growth phases as described above. In the presence of syringaldehyde, *C. beijerinckii* produced less protein, especially during stationary growth phase, when compared to growth in the absence of syringaldehyde as can be seen Table 2 and Fig. 5. Total protein levels of cell extracts of *C. beijerinckii* grown in control P2 and syringaldehyde-supplemented P2 media are the first evidence that syringaldehyde has a negative effect on protein concentration by *C. beijerinckii* strains. Following 24 h of fermentation, there is a 38% decrease in the amount of crude protein produced by *C. beijerinckii* grown in syringaldehyde-supplemented P2 medium compared to *C. beijerinckii* grown in control P2 medium (Table 2). At 36 h, there is a 52% decrease in total protein by *C. beijerinckii* grown in syringaldehyde-supplemented P2 medium compared to *C. beijerinckii* grown in control P2 medium. When total protein from equal amounts of *C. beijerinckii* cells grown in P2 and syringaldehyde-supplemented P2 medium media were extracted during acidogenic and solventogenic growth phases and applied onto SDS gel electrophoresis, band intensity of the crude proteins (Fig. 5) confirmed the results (decreased protein production by *C. beijerinckii* in the presence of syringaldehyde during solventogenic growth phase) obtained with Bradford protein assay method (Table 2). This indicates that syringaldehyde has a negative effect on protein concentration, including enzymes involved in ABE production pathway in *C. beijerinckii* strains. Interestingly, *C. beijerinckii* grown in syringaldehyde-supplemented P2 medium produced more protein than *C. beijerinckii* grown in control medium during initial stage (12 h) of fermentation (Table 2). The result may explain in part why *C. beijerinckii* had greater growth rate in the presence of syringaldehyde during the initial 24 h fermentation than the control fermentation.

**Table 2:** Total protein concentrations of cell-free extracts of *C. beijerinckii* NCIMB 8052 grown in the presence and the absence of syringaldehyde

Fermentation*	Fermentation Time (h)		
	12	24	36
Crude Protein (μg/mL)			
Control	58.75^a^ ± 1.12	59.93^c^ ± 2.93	63.34^e^ ± 2.62
Syringaldehyde-supplemented P2 medium	61.22^b^ ± 1.63	37.01^d^ ± 3.43	30.62^f^ ± 2.83

* Concentrations with the same superscript letter are not significantly different

However, we still do not know at this point why *C. beijerinckii* could not re-assimilate acetic and butyric acid from the medium in the presence of syringaldehyde which led to the accumulation of toxic levels of butyric and acetic acid in the fermentation medium (Fig. 2) and subsequent premature termination of the fermentation process. To gain some insights into this phenomenon, the effect of syringaldehyde on CoAT, was investigated. CoAT is an enzyme of interest in the metabolic pathway of solventogenic *Clostridium* species. CoAT is essential in the process of re-assimilating organic acid from the external environment and converting the acid to ABE. Any alteration in CoAT expression or activity levels would likely result in acid accumulation in the growth medium (Wiesenborn et al. 1989; Chen and Blaschek 1999). CoAT activity was assayed using cell-free extracts of *C. beijerinckii* grown in control P2 and syringaldehyde-supplemented P2 treatment media harvested after fermentation times of 12, 24 and 36 h respectively. These time points represent the acidogenic, early solventogenic, and mid-solventogenic growth phases (Mitchell 1998). Following 12 h of fermentation, CoAT activity from *C. beijerinckii* NCIMB 8052 cells grown in syringaldehyde-supplemented P2 medium exhibited about 76.3% of the activity obtained in the control (Table 3). The activity of CoAT from cells grown in syringaldehyde-supplemented P2 medium decreased further to only 26.8 and 17.2% of that of the control after 24 and 36 h fermentation, indicating that inhibition of CoAT expression in *C. beijerinckii* increased at fermentation time (24 h) close to the onset of solventogenesis (Table 3). This decrease in CoAT activity corresponds with the accumulation of acid in the syringaldehyde-supplemented P2 medium at 24 and 36 h (Fig. 3). With this level of CoAT expression, it becomes difficult for *C. beijerinckii* cells to effectively re-assimilate acetic and butyric acid. Taken together with the accumulation of levels of acid, the cell membrane functions no longer operate and result in premature termination of *C. beijerinckii* growth.

**Table 3:** CoA transferase activity level in cell-free extracts from *C. beijerinckii* NCIMB 8052

Treatment*	Fermentation time (h)*		
	12	24	36
Enzyme expression activity level (unit of activity/min/g cells)^y^			
P2 medium control	13.43^a^ ± 0.82	19.87^b^ ± 0.45	16.69^d^ ± 0.75
Syringaldehyde-supplemented P2 medium	10.25^a^ ± 0.76	5.32^c^ ± 0.95	2.87^e^ ± 0.30

^y^Equal amounts (2 mg dry weight) of *C. beijerinckii* 8052 cells grown in P2 medium (control) or syringaldehyde-supplemented P2 medium was used for the assay

* Activity levels with the same superscript letter are not significantly different

**Fig. 6:**
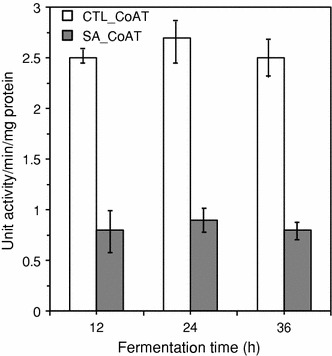
Effect of syringaldehyde on the activity of *C. beijerinckii* NCIMB 8052 CoAT. Cell-free extracts of *C. beijerinckii* were harvested following 12, 24, and 36 h of fermentation in control P2 medium. To obtain CTL_CoAT column, CoAT activity was assayed in the absence of syringaldehyde (control). For SA_CoAT column, syringaldehyde (1.0 g/L) was mixed CoAT prior to activity reaction assay

Furthermore, cell-free extracts of *C. beijerinckii* grown in control P2 medium harvested following fermentation times of 12, 24 and 36 h were assayed for CoAT activity in the presence of 1.0 g/L syringaldehyde. This assay was performed to determine in vitro if expressed CoAT experienced inhibition by syringaldehyde. In the presence of syringaldehyde, cell-free extracts of *C. beijerinckii* grown in control P2 medium and harvested at 12, 24 and 36 h fermentation had CoAT activities of 0.8, 0.9 and 0.8 units/min/mg protein, respectively (Fig. 6). This represents a 66–68% decrease in the activity when compared to the activity of the enzyme in the absence of syringaldehyde (Fig. 6), indicating that the activity of the expressed CoAT was negatively affected by syringaldehyde (Fig. 6). This suggests that syringaldehyde can negatively affect CoAT during ABE fermentation by *C. beijerinckii* in two ways, first by decreasing the expression during the growth (Table 3) and second by inhibiting the activity of CoAT following expression.

## Conclusion

The presence of inhibitory compounds in lignocellulosic hydrolysates will continue to have great influence on the fermentation of biomass to fuels and chemicals. The effect of lignocellulosic inhibitor (syringaldehyde) on the growth and ABE production by solventogenic *C. beijerinckii* NCIMB 8052 was investigated. Expression of CoAT by *C. beijerinckii* during growth in the presence of syringaldehyde was lower than that obtained during growth in the absence of syringaldehyde. The activity of expressed CoAT, in addition, was negatively affected by syringaldehyde. These inhibitory effects on the CoAT system of *C. beijerinckii* hampered the ability of this microorganism to convert acetic and butyric acid to neutral ABE. The accumulation of butyric and acetic acid led to the premature termination of the fermentation process.
